# *Miscanthus* x *giganteus* Stem Versus Leaf-Derived Lignins Differing in Monolignol Ratio and Linkage

**DOI:** 10.3390/ijms20051200

**Published:** 2019-03-09

**Authors:** Michel Bergs, Georg Völkering, Thorsten Kraska, Ralf Pude, Xuan Tung Do, Peter Kusch, Yulia Monakhova, Christopher Konow, Margit Schulze

**Affiliations:** 1 Department of Natural Sciences, Bonn-Rhein-Sieg University of Applied Sciences, von-Liebig-Strasse 20, D-53359 Rheinbach, Germany; michel.bergs@h-brs.de (M.B.); xuan-tung.do@h-brs.de (X.T.D.); peter.kusch@h-brs.de (P.K.); 2 Institute of Crop Science and Resource Conservation, Faculty of Agriculture, University of Bonn, Klein-Altendorf 2, D-53359 Rheinbach, Germany; g.voelkering@uni-bonn.de (G.V.); r.pude@uni-bonn.de (R.P.); 3 Field Lab Campus Klein-Altendorf, Faculty of Agriculture, University of Bonn, Campus Klein-Altendorf 1, D-53359 Rheinbach, Germany; kraska@uni-bonn.de; 4 Spectral Service AG, Emil-Hoffmann-Strasse 33, D-50996 Köln, Germany; monakhova@spectral-service.de; 5 Institute of Chemistry, Saratov State University, Astrakhanskaya Street 83, 410012 Saratov, Russia; 6 Department of Chemistry, MS 015, Brandeis University, 415 South Street, Waltham, MA 02453, USA; ckonow@brandeis.edu

**Keywords:** biomass, chemometrics, genotype, HSQC NMR, lignin, *Miscanthus* x *giganteus*, monolignol ratio, principal component analysis

## Abstract

As a renewable, *Miscanthus* offers numerous advantages such as high photosynthesis activity (as a C_4_ plant) and an exceptional CO_2_ fixation rate. These properties make *Miscanthus* very attractive for industrial exploitation, such as lignin generation. In this paper, we present a systematic study analyzing the correlation of the lignin structure with the *Miscanthus* genotype and plant portion (stem versus leaf). Specifically, the ratio of the three monolignols and corresponding building blocks as well as the linkages formed between the units have been studied. The lignin amount has been determined for *M*. x *giganteus* (Gig17, Gig34, Gig35), *M*. *nagara* (NagG10), *M*. *sinensis* (Sin2), and *M*. *robustus* (Rob4) harvested at different time points (September, December, and April). The influence of the *Miscanthus* genotype and plant component (leaf vs. stem) has been studied to develop corresponding structure-property relationships (i.e., correlations in molecular weight, polydispersity, and decomposition temperature). Lignin isolation was performed using non-catalyzed organosolv pulping and the structure analysis includes compositional analysis, Fourier transform infradred (FTIR), ultraviolet/visible (UV-Vis), hetero-nuclear single quantum correlation nuclear magnetic resonsnce (HSQC-NMR), thermogravimetric analysis (TGA), and pyrolysis gaschromatography/mass spectrometry (GC/MS). Structural differences were found for stem and leaf-derived lignins. Compared to beech wood lignins, *Miscanthus* lignins possess lower molecular weight and narrow polydispersities (<1.5 *Miscanthus* vs. >2.5 beech) corresponding to improved homogeneity. In addition to conventional univariate analysis of FTIR spectra, multivariate chemometrics revealed distinct differences for aromatic in-plane deformations of stem versus leaf-derived lignins. These results emphasize the potential of *Miscanthus* as a low-input resource and a *Miscanthus*-derived lignin as promising agricultural feedstock.

## 1. Introduction

*Miscanthus*, a perennial rhizome-forming sweet grass (*Poaceae*), is originally cultivated in subtropical regions in Asia. Currently, there are approximately 17 species investigated in Europe for energy and material development including *Miscanthus tinctorius*, *M. sinensis, M. sacchariflorus*, and the triploid genotype *M.* x *giganteus,* which is an allotriploid hybrid of the diploid *M. sinensis* and the tetraploid *M. sacchariflorus. Miscanthus* field trials have been performed in Europe over the past 25 years confirming that *M.* x *giganteus* hybrids show high yields over a wide range of environmental conditions [1,2,3]. As a C_4_ plant, Miscanthus is effective in carbon sequestration due to the formation of oxaloacetate, as opposed to D-3-phosphoglycerate for C_3_ plants [4,5,6]. Thus, Miscanthus is one of the very few crops that is studied to serve as a CO_2_ sink [7,8,9,10,11,12,13]. In addition, this sterile genotype does not generate fertile seeds, which avoids uncontrolled spreading [14]. After planting, the production of the perennial crop lasts for about 20 years. In the production phase, the plant needs much less fertilizer than other crops, even at arid conditions, due to an effective nitrogen metabolism. Plant protection is, in general, not required except for weed control in the first year of establishment [2,15,16]. *M.* x *giganteus* reaches a height of about four meters and an annual production up to 25 t/ha, which saves production costs that decrease the environmental impact due to reduced tillage and less soil compaction and erosion [17]. Within the last five years, the upscaling of Miscanthus biomass production in Europe could significantly be improved by using seed-based hybrids. Consequently, *Miscanthus* hybrids are expected to play an increasing role in the provision of perennial lingo-cellulosic feedstock in Europe, which contributes to a lower carbon economy [1,18,19].

The use of *Miscanthus* as a source for raw materials would significantly improve the environmental performance of lignocellulose-based biorefineries [20,21,22]. *Miscanthus* is already used for direct combustion in a sustainable and CO_2_ neutral manner. In addition to the exploitation of *Miscanthus* as an energy plant, there is urgent need to exploit the potential of *Miscanthus* for chemical production. Recent work by Kraska et al. has shown *Miscanthus’* potential for chemical production through a cascade utilization [23,24]. Other studies focus on the production of bioethanol [25], hydrogen [26], and the utilization of the plant fibers for fiber-based composites [27,28,29,30,31].

So far, only a few systematic studies have been published focusing on the structure-property relationships for *Miscanthus*-derived lignins. El Hage et al. reported the pre-treatment of biomass for ethanol-based organsolv pulping, in which they specify correlations of structural changes with process conditions [32,33,34]. Catalyzed organosolv pulping was studied to depolymerize lignins using vanadium and nickel-based catalysts, which shows correlations between lignin structure and reactivity toward the catalysts [35,36,37]. Timilsena et al. studied *Miscanthus* plants and compared different pre-treatment methods (i.e., autohydrolysis, 2-naphthol treatment) [38]. Other authors studied different Miscanthus genotypes regarding their composition (cellulose, hemicellulose, lignin), but did not analyze the lignin structure [39]. Van der Weijde investigated eight M. *sinensis* samples regarding their cell wall constitution [40]. Enzymatic depolymerization of Miscanthus-derived lignin was studied by various groups [41,42,43].

The most detailed study was reported by Da Costa et al., which investigated the cell tissue type of 25 *Miscanthus* genotypes (i.e., *M*. x *giganteus*, *M*. *sinensis*, and *M*. *sacchariflorus*) [44]. Cell-wall compositional differences between *Miscanthus* stem and leaf samples were mainly associated with differences of structural carbohydrates. The authors could proof direct correlations between the lignin content isolated form specific plant portions and the genotype. However, no structural details regarding the monolignol ratio and/or corresponding linkages were reported [44]. 

Iqbal et al. reported a multi-genotype trial performed in south Germany focusing correlations of biomass yield and composition with climatic conditions and harvesting time using multivariate data analysis methods [45]. For example, in *M*. *sacchariflorus*, the harvest date–aging interaction influenced the Mg concentration and ash content. The harvest date–rainfall interaction significantly decreased calcium, silicon, and nitrogen concentrations. In addition, these deviations in mineral concentration depend on the *Miscanthus* genotypes (as studied for *M*. *x giganteus* and *M*. *sinensis*). This study did not include lignin 3D structure analysis [45]. 

**Figure 1:**
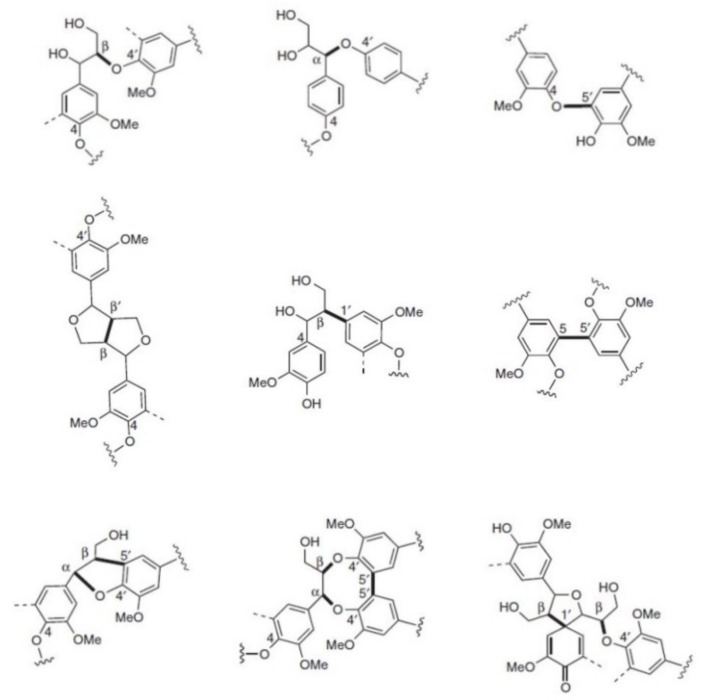
Monolignol linkages. First line: Ether bonds (ß-O-4’, α-O-4’, 4-O-5’). Second line: C-C-bonds. (ß-ß’, ß-1’, 5-5’) and third line: more complex linkages (ß-5’/ α-O-4’, 5-5’/ ß-O-4’/ α-O-4’, ß-1’/ ß-O-4’).

In contrast to the lack of a systematic structural study for *Miscanthus*-derived lignins, numerous studies are reported regarding the structure of wood-derived kraft lignin obtained from industrial black liquor. In this scenario, HSQC 2D-NMR is one of the most widely used tool for structural characterization of lignins of any origin, which is comprehensively reviewed by Ralph, Lupoi, and Tarasov et al. [46,47,48]. Most interestingly, HSQC provides information on the units (G, H, S) and corresponding inter-unit linkages. Depending on the biomass source (soft and hard wood, grasses) and the pulping process (kraft, steam explosion, organosolv etc.), the monolignol ratio, and their linkages differ significantly [49,50,51,52,53,54,55,56,57,58,59,60]. Figure 1 shows the most common linkages of lignin formed during biosynthesis in which some of them were confirmed within the last five years [55,61,62].

Very recently, Lan et al. reported the synthesis of several 4’–O–ß-coupling products of tricin with coniferyl and sinapyl alcohol and revealed new correlations in the HSQC spectrum [63]. Table 1 gives an overview about differences regarding the linkages found for hard wood, soft wood, and *Miscanthus* grasses [61,62,63].

In addition, the combination of NMR and chemometrics has been successfully applied in the origin analysis of biopolymers including polysaccharides and lignin. For example, Monakhova et al. were recently able to specify porcine and ovine-derived heparins using 2D DOSY NMR and size exclusion chromatography (SEC) data [64]. In general, chemometric methods can also be used for multivariate FTIR data analysis. Boeriu et al. combined FTIR and principal component analysis (PCA) to be used as a fast and reliable non-destructive technique to classify the botanical origin of lignins. In addition, chemometric models (partial least squares, PLS regression) allowed an accurate determination of phenolic hydroxyl groups and corresponding antioxidant capacity [65]. Chen et al. used multivariate data analysis to characterize wood samples qualitatively and quantitatively by FTIR spectroscopy. To determine the contents of lignin, cellulose, and hemicellulose, respectively, the authors included a broad variety of input parameters such as wood species obtaining results with the root-mean-square errors of 1.51%, 0.96%, and 0.62% [66]. Very recently, Christou et al. reported a study examining the combination of FTIR spectroscopy and chemometrics to differentiate carob samples from seven different Mediterranean countries using the first derivatives of the FTIR spectra, which resulted in a confidence level up to 95% [67].

For the first time, this study presents comprehensive analytical data (including compositional analysis, FTIR, UV-Vis, HSQC-NMR, SEC, TGA, Py-GC/MS) for *Miscanthus* x *giganteus*-derived lignins correlating with plant portions (stem versus leaf). The biomass was separated into stem and leaf portions to be treated separately using the organosolv process for lignin isolation. In addition to conventional analysis of the chemical structure of the obtained lignins, multivariate data analysis of FTIR data is used to specify structural details that allow the discrimination of stem versus leaf-derived lignins. 

## 2. Results and Discussion

### 2.1. Miscanthus Crop Analysis

**Figure 2:**
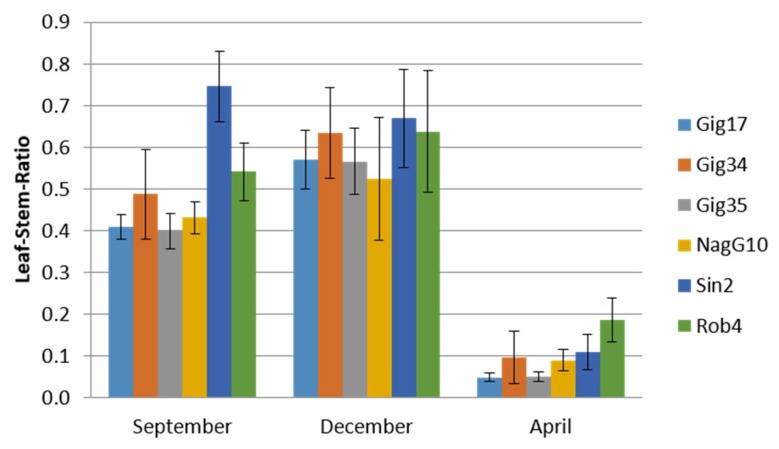
*Miscanthus* crop analysis via NREL measurements. Leaf versus stem content of *Miscanthus* genotypes: *M*. x *giganteus* (Gig17, Gig34, Gig35), *M*. *nagara* (NagG10), *M*. *sinensis* (Sin2), and *M*. *robustus* (Rob4) harvested in September (09/15), December (12/14), and April (04/15), respectively (arranged to follow the seasonal order from autumn to spring).

The seasonal influence on plant constitution was examined. Stem and leaf samples for lignin analysis were harvested in December 2014 as well as in April and September 2015 to compare across different years and harvest times. Plant constitution analysis showed significant differences regarding the lignin amount with respect to the year and harvest date (Figure 2). In early autumn (September), the leaf content did not reach the maximum (except for *M*. *sinensis*) when compared to the December harvest. Until spring, the plants lose their leaves, which results in an extremely low leaf versus stem ratio. The highest values were found for *M*. *robustus* (Rob4).

In the following, the leaf-derived and stem-derived lignins will be discussed in detail for two *M.* x *giganteus* cultivars (Gig17, Gig34). Special focus is given to similarities and differences in lignin content and monolignol constitution depending on the plant portion (stem vs. leaf). 

### 2.2. NREL Compositional Analysis

Four samples were analyzed regarding their element constitution following the NREL protocol. Both *M.* x *giganteus* samples were harvested in April 2015 and were then separated into stem and leaf portions to be analyzed separately (Table 2). 

The total ash content between stem-derived and leaf-derived lignins differs. Average values for stem lignins are 2.58% vs. 5.71% for leaf lignins due to a higher mineral content in leaves. Wahid et. al. reported total ash contents for stems of 2.6%–4.0% (*M*. x *giganteus*) and 1.5%–3.4% (*M*. *sinensis*). Leaves varied between 3.9%–7.2% (*M*. x *giganteus*) and 3.3%–5.0% (*M*. *sinensis*) [68]. Acid-insoluble residuals (AIR) showed very low ash contents (average 0.94%, calculated for all six genotype samples listed in Figure 2, [69]) due to low mineral impurities in this fraction. Due to a higher cellulose content in stems compared to leaves, higher glucose values were obtained for both *M*. x *giganteus* samples. In literature, carbohydrate contents vary in the range of 40 to 50 wt % for *Miscanthus* x *giganteus* genotypes, mainly consisting of glucan and xylan [70]. Other sugars, i.e., arabinan, mannan, and galactan are usually below 2%, which was very recently reported by Scaglione-Mellor for different *Miscanthus* genotypes [71]. No significant differences were found for the total lignin content for leaf and stem-derived biomasses. In literature, Miscanthus-derived lignin contents vary in the range of 19–25%. This mainly depends on the plant genotype [72,73].

### 2.3. FTIR Spectroscopy

**Figure 3:**
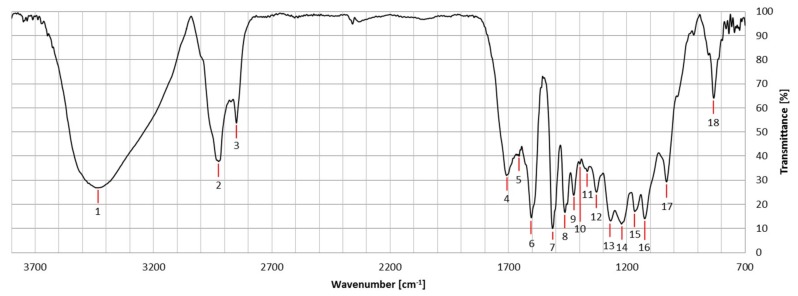
FT-IR spectra of Gig17 (stem, leaf, and mixture) with numbered signals listed in Table 4.

The results of the FTIR analysis confirmed reported literature data for lignins obtained from *M*. x *giganteus* plants [74,75]. As an example of the results, Table 3 summarizes the FTIR signals of sample Gig17, which is an *M*. x *giganteus* stem/leaf mixture. Signal numbers are given in Figure 3.

**Figure 4:**
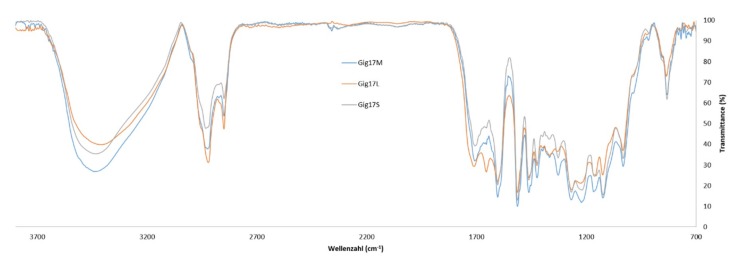
FT-IR spectra of mixture (Gig17M), leaf (Gig17L), and stem-derived (Gig17S) lignins obtained from *M.* x *giganteus* (Gig17).

**Figure 5:**
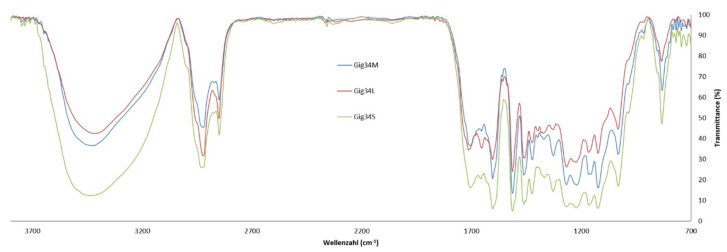
FT-IR spectra of mixture (Gig34M), leaf (Gig34L), and stem-derived (Gig34S) lignins obtained from *M*. x *giganteus* (Gig34).

Comparing the FTIR data of stem/leaf mixtures (Gig17M) versus separated biomass (Gig17L, Gig17S), similarities were found for mixture and stem-derived lignin (Figure 4 and Figure 5).

This is in good accordance with the low amount of leaves in the plant (<5 wt %). In detail, both leaf samples showed a signal at about 1655 cm^−1^ (number 5), which is very weak for stem (Gig17S, Gig34S) and the mixtures (Gig17M, Gig34M). Further differences are obtained for signal No. 12 (ca. 1331 cm^−1^), No. 14 (ca. 1225 cm^−1^), and 16 (ca. 1124 cm^−1^). All three are very weak for leaf-derived lignin versus stem and mixture, respectively. The two *M.* x *giganteus* cultivars (17 and 34) showed no significant differences.

**Figure 6:**
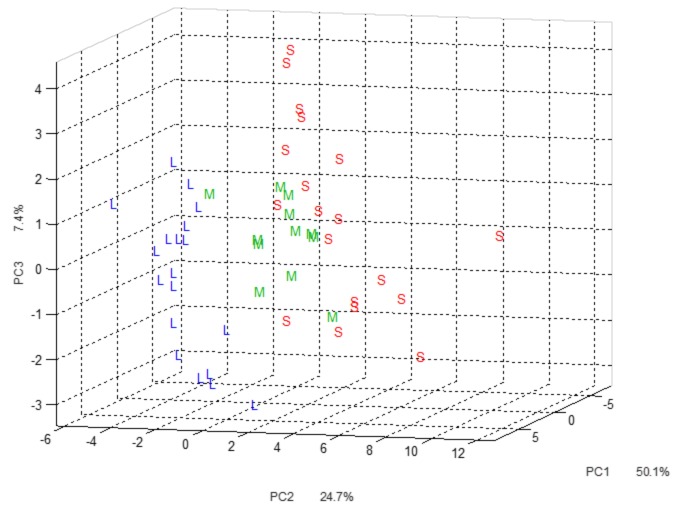
Multivariate data analysis of FT-IR data using the principal component analysis (PCA). L: leaf-derived lignin. M: mixture-derived lignin. S: stem-derived lignin.

PCA characterization of lignin samples was carried out to evaluate the potential of multivariate data analysis to specify lignin obtained from *Miscanthus* x *giganteus* plants derived from stems, leaves, and their mixtures. The projections of IR spectra of lignin samples on the first three principal components (PCs), which express 82% of variance, are shown in Figure 6.

The first two PCs are enough to differentiate steam-derived and leaf-derived materials, whereas PC3 is necessary to highlight the third group of samples (mixtures). The mixture class (M) is completely separated from the leaf class (L), but still shows overlap with the stem class (S). Again, this is most likely due to the low ratio of leaf versus stem lignin in the mixture. Advanced chemometric methods such as discriminant analysis are required to separate all three groups in the multivariate space. Loadings plots confirmed our univariate findings described above.

### 2.4. Size Exclusion Chromatography

**Figure 7:**
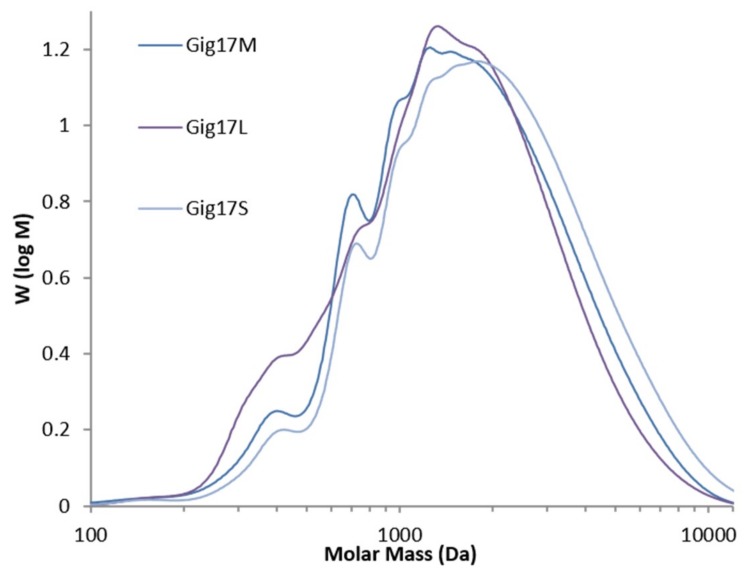
SEC curves of lignins obtained from *Miscanthus* x *giganteus* (Mixture: Gig17M. Leaf: Gig17L. Stem Gig17S).

Analogous to the FTIR results, SEC studies revealed distinct differences between stem-derived and leaf-derived lignins and similarities for stem/leaf mixtures and stem lignins. In detail, the SEC curve profile differs for leaf-derived lignin, which shows weak shoulders at molar mass below 1000 Da instead of small but sharp peaks for stem-derived and mixture-derived lignin (Figure 7).

All obtained data (number and weight average molar masses (*M*_n_, *M*_w_) and polydispersities (PD) are summarized in Table 4.

There are weak deviations for *M*_n_ and *M*_w_ in stem versus leaf lignin. However, these differences did not influence the final polydispersity, which is very narrow for all samples. Literature data for organosolv-derived *Miscanthus* lignin vary between 2.0 to 2.5 kDa (*M*_w_), 1.1 to 1.6 kDa (*M*_n_), and 1.5 to 2.0 (PD) [76]. This is a much lower PD than beech wood-derived organosolv lignins, which showed polydispersities of 2.7 to 2.9 [47,77,78]. Brandt et al. reported SEC data for *M*. x *giganteus*-derived lignin isolated via ionic solvents showed significantly higher polydispersities of 3.7–7.6 depending on pre-treatment times [79]. The *M*. x *giganteus* samples isolated via organosolv treatment showed the narrowest polydispersity and the resulting macromolecular homogeneity reported so far for grass-derived lignins.

Structural differences for stem-derived versus leaf-derived lignins, namely lower molecular weight of the leaf-lignins, could also be confirmed by spectroscopic methods (FTIR, UV-Vis, see Section 2.3 and Section 2.5).

### 2.5. UV-Vis Spectroscopy

**Figure 8:**
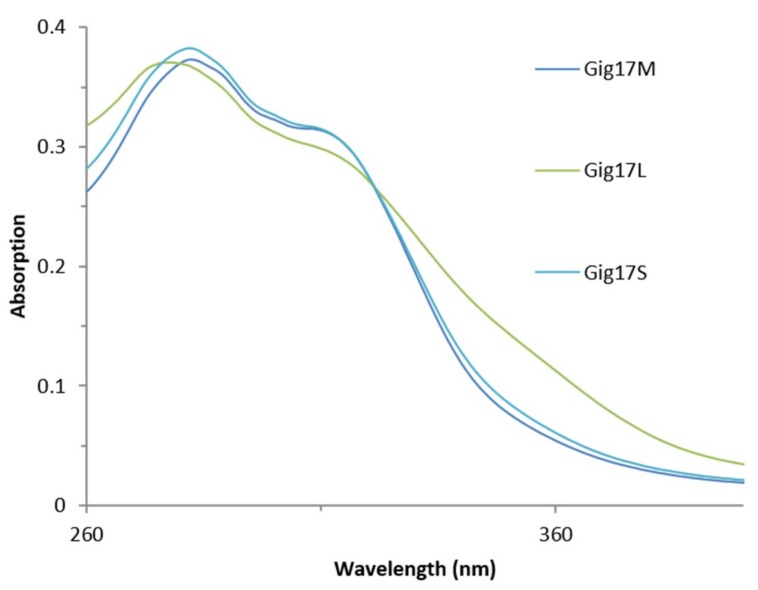
UV/Vis curves for lignins obtained from *Miscanthus* x *giganteus.* (Mixture: Gig17M. Leaf: Gig17L. Stem Gig17S).

As shown in Figure 8, *M*. x *giganteus* lignin obtained via organosolv pulping samples showed two distinct signals, which is in accordance to literature data [35].

Again (due to the much higher amount of stems in the mixture), mixtures (Gig17M) and stem-derived lignin (Gig17S) are comparable in their UV absorption profile, whereas leaf-derived lignin (Gig17L) differ in their UV-Vis absorption behavior. In particular, leaf-lignin consists of higher amounts of conjugated fragments resulting in a weak but visible shoulder around 320 to 350 nm. This is in accordance with the absorption profile of lignin from other biomasses [22,32,47,80]. Apart from this, four distinct absorption maxima were found for kraft lignins obtained from technical black liquor (spruce/pine mixtures) and purified via sequential extraction from organic solvents. Clearly, stepwise purification influenced the intensity of UV/Vis absorption signals (in addition to effects caused by biomass and the pulping process) [80]. 

### 2.6. Thermogravimetric Analysis

The thermal behavior of the lignins was determined by thermogravimetric analysis (TGA). In Table 5, the TGA data obtained in this study are summarized including time-depending mass loss (in %/min), residual masses, and decomposition temperatures for mixtures (Gig17M) and leaf/stem-separated samples (Gig17L, Gig17S).

The first mass loss at about 120 °C (after 5 min) can be assigned to water elimination. Subsequently, lignin was systematically depolymerized between 170–350 °C (starting after ca. 8 min). Corresponding decomposition temperatures vary between 335–380 °C with the lowest values for leaf-derived lignins. However, the data obtained for Td and residual mass differed by less than 10% and no clear correlation could be drawn between the thermal behavior and plant portion and/or *Miscanthus* genotype. Most likely, differences in sample pre-treatment and preparation are of more significant influence, which confirms results reported in the literature [81,82].

In general, lignins are thermally degraded in a broad temperature range (100–700 °C) due to their rather complex 3D structure consisting of phenolic hydroxyl, carbonyl, and benzylic hydroxyl functionalities resulting in several weight-loss steps during TGA measurements. The first loss (between 100–200 °C) can be attributable to moisture and other volatile substances such as carbon monoxide and dioxide, which confirms literature data [83]. According to Wittkowski et al., the degradation of propanoid side chains occurs between 230 to 260 °C, which results in the splitting of methyl, ethyl, and vinyl guaiacol derivatives, whereas aryl ether bonds are usually split below 310 °C [84]. In most lignins, about two-thirds of the monolignol units are connected by β-O-4’ linkages, which results in decomposition temperatures varying from 350 to 425 °C for lignin for all analyzed sources of biomass (soft and hard wood, grasses, LCF-rich waste) [47]. Glasser and El-Saied reported a correlation of decomposition temperature and a monolignol ratio. Lignins with higher contents of G units and a corresponding high amount of very stable intermolecular C–C bonds show higher decomposition temperatures [85,86].

### 2.7. Pyrolysis Gas Chromatography/Mass Spectrometry

**Figure 9:**
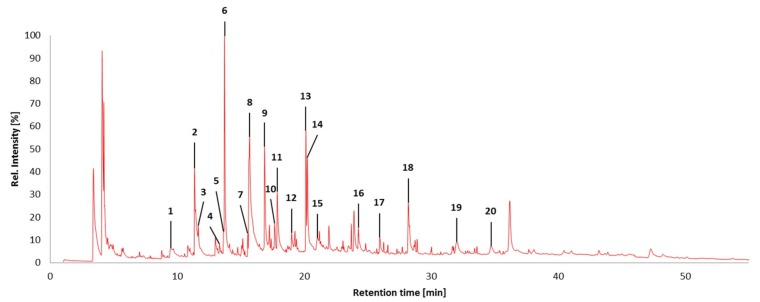
Pyrolysis-GC/MS pyrogram of a lignin obtained from *Miscanthus* x *giganteus* Gig17M. Stem/leaf mixture with numbers for all aromatic fragments listed and assigned in Table 6.

Pyrolysis GC/MS analysis has indicated significant variations in monolignol ratios between stem, leaf, and mixed samples. In this case, the assignment of the Py-GC/MS data was performed in accordance with data reported by Hodgson et al. [87,88]. Figure 9 shows a typical pyrogram (obtained for Gig17M). All aromatic pyrolysis products are numbered and summarized in Table 6.

**Figure 10:**
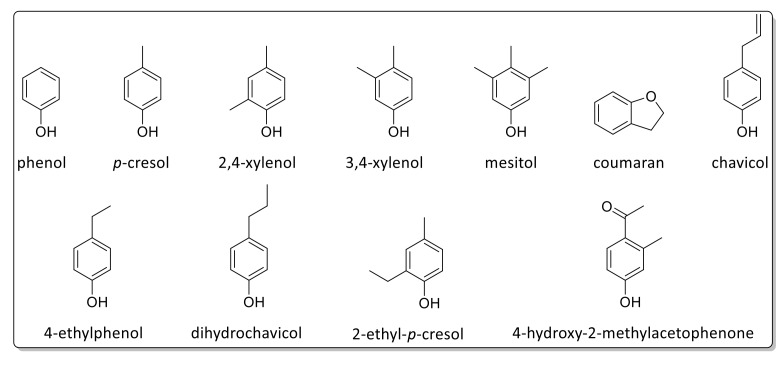
Detected Py-GC/MS fragments attributed to the H-unit.

**Figure 11:**
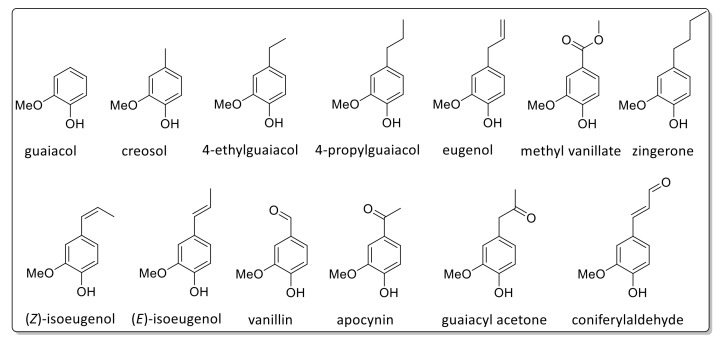
Detected Py-GC/MS fragments attributed to the G-unit.

**Figure 12:**
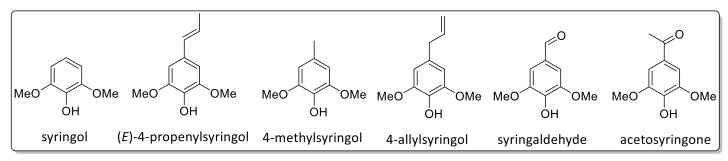
Detected Py-GC/MS fragments attributed to the S-unit.

A few signals could not be attributed to a certain monolignol unit. Based on these data, monolignol ratios were estimated (Figure 10, Figure 11 and Figure 12).

As summarized in Table 7, Py-GC/MS fragments assigned to the syringyl (S) unit showed the lowest values for both leaf-derived lignin samples whereas the highest values were found for p-hydroxyphenyl (H) units. However, it should be emphasized that this is merely the first estimation since not all Py-GC/MS data could be assigned. In general, all samples investigated in this study showed a high amount of H units (Table 7), which confirms data reported by Brandt et al. for lignin isolated from *M*. x *giganteus* using ionic liquids [78]. 

According to Py-GC-MS analysis, fragments released from untreated *Miscanthus* originated from H/*p*-coumaric acid, G/ferulic acid, and S in roughly equal amounts (32%, 35%, and 33%, respectively). For quantification in pyrolysis gas chromatography, FID detectors are used and give nearly the same response as MS detectors once the compounds have been identified [89]. The application of the MS detector for the quantification and determination of the monolignol ratio of lignins in our work could be the cause of the discrepancy between the quantitative results obtained from Py-GC/MS and HSQC-NMR methods (see next Section 2.8).

### 2.8. Nuclear Magnetic Resonance

Heteronuclear single quantum correlation (HSQC) 2D-NMR was used to characterize the structure of *M.* x *giganteus* derived lignin with a particular focus on monolignol ratio and corresponding linkages. In accordance with literature data, cell wall and lignin HSQC spectra can be divided into the following regions: the aliphatic (side chain) and the aromatic (core) region. The aliphatic region does not provide structural information except for acetyl substituents and/or hemi-cellulosic component. Table 8 summarizes the obtained HSQC data.

**Figure 13:**
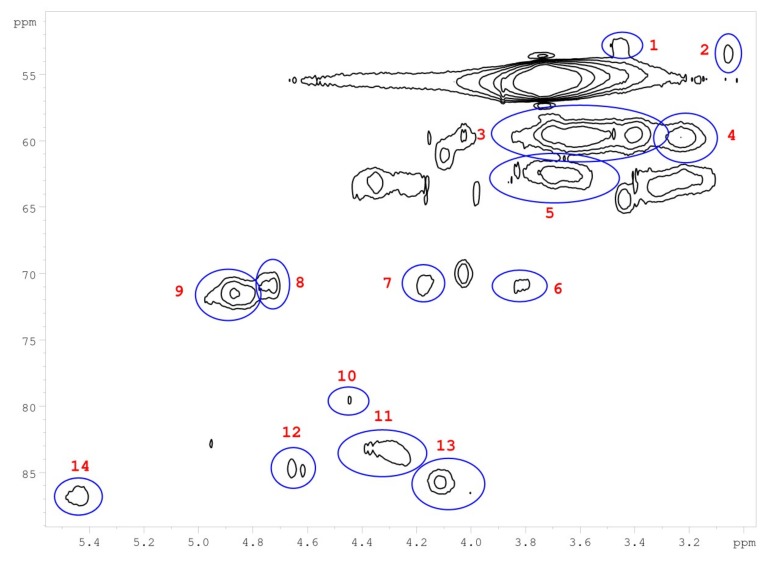
Non-aromatic HSQC region of a lignin obtained from *M.* x *giganteus* stem/leaf mixture (Gig17M). Numbers are listed and assigned in Table 9.

**Figure 14:**
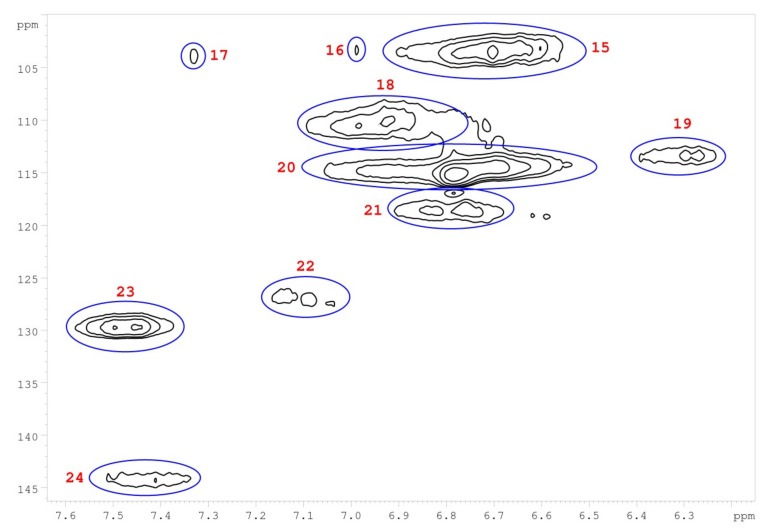
Aromatic HSQC region of a lignin obtained from *M.* x *giganteus* stem/leaf mixture (Gig17M). Numbers are listed and assigned in Table 9.

Integral numbers 1–14 found in the non-aromatic region and attributed monolignol linkages (Figure 13). Integrals 15–24 were obtained in the aromatic region assigned to the monolignol units (H, G, S) (Figure 14).

Corresponding ratios of monolignols and linkages were estimated. As summarized in Table 9, the data show differences between mixtures and lignin obtained from a separated stem versus a leaf. In leaves, about two-thirds of the G unit was found, whereas stem and mixture samples showed rather low G content. Instead, H and S units are at a higher ratio in mixtures.

**Figure 15:**
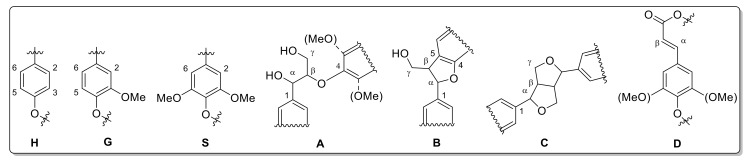
Lignin structure elements for HSQC NMR signal assignment (A: β-O-4 linkage, B: phenylcoumaran, C: resinol, D: β-unsaturated ester).

However, HSQC NMR only considers signals for free C-H coupling (in case of further aromatic substituents, those fragments would not be recognized) [48,63,90]. Therefore, further detailed analysis is required to explain deviations of the monolignol ratios obtained via pyrolysis GC/MS and HSQC-NMR. Based on the HSQC data, the ratio of most abundant monolignol linkages was estimated (Table 10 and Figure 15).

In accordance with reported data for *Miscanthus*-based lignins, β-arylether were most abundant linkages, which vary between 55% and 65%. About 10% of the signals could be assigned to phenylcoumarane and resinol linkages. Unsaturated esters (D) are most abundant in stem-based lignins (up to 30%). Contrary to literature data, no signals were found for residual carbohydrates confirming the purity of the lignins obtained via organsolv pulping. 

## 3. Materials and Methods 

### 3.1. Miscanthus Field Trial Lignin Isolation via Organosolv Pulping

*Miscanthus* genotypes used in this study are cultivated at the field lab Campus Klein-Altendorf (University of Bonn, Rheinbach, Germany). The field stand was established in 2012. Genotypes used in this study were *M*. x *giganteus* (cultivars 17, 34, and 35, named as Gig17, Gig34, Gig35), *M*. *nagara* (NagG10), *M*. *sinensis* (cultivar 2, named Sin2), and M. *robustus* (cultivar 4, named Rob4, a hybrid of *M*. *sacchariflorus* and *M*. *sinensis*) [15,16]. *Miscanthus* samples were taken from the field stand in December 2014, April 2015, and September 2015.

### 3.2. Lignin Isolation via Organosolv Pulping

The samples were milled (using a ball mill Pulverisette 6, Fritsch, Idar-Oberstein, Germany) and sieved (using a Modell AS 200 basic, Retsch, Haan, Germany) to a particle size <0.5 mm. The organosolv process was performed according to an earlier published procedure [34,91,92,93]. All samples were prepared without using catalysts. Approximately 50 g *Miscanthus* x *giganteus* was passed through a 0.5 mm sieve and then mixed with 400 mL Ethanol (80% υ/υ). The mixture was heated at 170 °C for 90 min under continuous stirring in a Parr reactor with a Parr 4848 Reactor Controller. Afterwards, the *Miscanthus* biomass is vacuum filtrated and washed 5 times with 50 mL Ethanol (80% υ/υ). Three volumes of water and approximately 10 mL hydrochloric acid was added to the filtrate to precipitate the organosolv lignin, which was collected by centrifugation at 3500 rpm for 5 min and washed 3 times with distilled water. Lastly, the samples were freeze-dried for 72 h.

### 3.3. Lignin Purity, Ash, and Sugar Content via NREL Measurements

The chemical composition (%, *w*/*w*) was determined according to the standard analytical procedures published by the National Renewable Energy Laboratory (NREL) (Determination of Structural Carbohydrates and Lignin in Biomass) [94]. NREL measurements were performed by the BIOPOS Research Institute (Teltow-Seehof, Germany). HPLC analysis was conducted using water at a flow rate of 0.4 mL/min in a column (300 × 7.8 mm, Machery-Nagel, Bethlehem, PA, USA) at a constant temperature of 90 °C. Shortly, structural carbohydrates in biomass and lignin samples were determined following NREL/TP-510-42618 [95], biomass extracts in water and ethanol, according to NREL/TP-510-42619 [96], dry matter in biomass according to NREL/TP-510-42621 (Sluiter et al., 2008) [97] and ash contents according to NREL/TP-510-42622 [98].

### 3.4. Size-Exclusion Chromatography

To evaluate the depolymerization of the lignins, SEC was performed using the parameters shown in Table 11. Samples with a concentration of 10 mg·mL^−1^ were prepared in tetrahydrofuran (THF) and filtrated through 0.2 µm PTFE filter prior to analysis. For molar weight evaluation, polystyrene was used as a calibration standard.

Size exclusion chromatography was performed using a PSS SECurity2 GPC System, Mainz, Germany (UV detector, 280 nm) to determine the weight-average (*M*_w_) and number-average (*M*_n_) molecular weights of the lignins as well as their polydispersity (PD). Tetrahydrofuran (THF) was the mobile phase with a run time of 30 min and an injection volume of 60 µL and polystyrene calibration standards. The lignin sample was completely dissolved in THF (1 mg·mL^−1^) with gentle stirring at room temperature.

### 3.5. UV-Vis Spectroscopy

The lignin UV-Vis absorption was measured using a Hewlett-Packard (Waltham, MA, USA) 450 Diode Array spectrometer. Samples of 1 mL containing 0.5 mg·mL^−1^ lignin in THF were measured at room temperature in a range of 260 to 400 nm.

### 3.6. Fourier Transform Infrared Spectroscopy

FTIR-spectra were recorded in a wavenumber range of 400–700 cm^−1^ with a resolution of 4 cm^−1^ and eight scans per sample (Jasco, Tokyo, Japan). KBr disc containing 2 mg of sample and 300 mg of KBr were prepared using a hydraulic press (10 metric tons).

### 3.7. Multivariate Data Analysis

Multivariate data analysis was performed using principal component analysis (PCA) in MATLAB 2019a (The Math Works, Natick, MA, USA). SAISIR package for MATLAB was used for statistical calculations [99,100]. The data points from IR spectral regions (800–1’800 and 2’800–3’700 cm^−1^) were selected. Autoscaling was used as the pretreatment before PCA. To normalize the intensities in different samples, they were additionally scaled to the total intensity.

### 3.8. Nuclear Magnetic Resonance Spectroscopy

2D HSQC NMR spectra were recorded using a NMR spectrometer Avance III 600 (Bruker, Karlsruhe, Germany) with 4 scans and 16 prior dummy scans. Deuterated dimethyl sulfoxide (DMSO d-6) was used as the solvent, tetramethylsilane as the standard, and 100 mg/mL as the sample concentration. A spectral width of 7211 Hz was used, resulting in 4000 points (receiver gain of 2050). A total acquisition time of 0.28 s. O1 was set to 5 ppm (^1^H) and 80 ppm (^13^C).

### 3.9. Thermogravimetric Analysis

Approximately 10 mg of lignin were used for thermogravimetric analysis (TGA) using a Netzsch (Selb, Germany) TGA 209 F1 (at a heating rate of 20 °C·min^−1^, nitrogen atmosphere). The temperature ranged from an ambient temperature to 900 °C. At 900 °C, synthetic air was used for an isothermic combustion.

### 3.10. Pyrolysis-Gas Chromatography/Mass Spectrometry

Pyrolysis-GC/MS measurements were performed on a combination of two GC 2010 Plus gas chromatographs, a GCMS-QP2010 Ultra mass spectrometer (Shimadzu, Kyoto, Japan), and an EGA/PY-3030D multi-shot pyrolyzer (Frontier Lab, Fukushima, Japan). The two fused silica capillary columns were Ultra Alloy columns (UA5-30M-0.25F and UACW-20M-0.25F) with a length of 30 m each, an inside diameter of 0.25 mm, and a film thickness of 0.25 μm. Shimadzu software MDGC Analysis GC solution 2.41.00.SU1 was used for the measurement and Shimadzu GC/MS solution 2.72 for the evaluation [73]. A 0.5 mg lignin sample was placed in a pyrolysis crucible made of stainless steel (PY1-EC50F), inserted into the pyrolyzer, and pyrolysed at 550 °C. For the first capillary column, the following temperature program was used: 75 °C for 1 min and 280 °C for 25 min at a heating rate of 7 °C·min^−1^. The column of the second GC instrument was held isothermal at 200 °C. Helium, grade 4.0 (99.990 %, *v*/*v*) (Westfalen AG, Münster, Germany) was used as carrier gas. After GC separation of the pyrolysis products, the MS detection was carried out. The ionization of the substances took place via electron impact ionization (EI) at 70 eV. The substances were accelerated in a high vacuum by the electric field of the quadrupole, separated, and then detected by their mass/charge ratio (m/z) in the electron multiplier. The *m*/*z* measuring range was 35–740 u.

## 4. Conclusions

This study presents the first systematic analysis of lignin amount and 3D structure for two different *M*. x *giganteus* genotypes (Gig17, Gig34) separated into stem and leaf portions. For biomass pulping, the organosolv process was used. Depending on the plant component (leaf vs. stem), structure-property-relationships have been developed. According to SEC analysis, lignin molecular weights and polydispersities differ for stem and leaf-derived lignins. Spectroscopic data (HSQC-NMR) revealed differences in the monolignol ratio and linkages for stem-based and leaf-based lignins. Further studies are required to explain deviations of pyrolysis GC/MS and HSQC-NMR results. FTIR data discrimination was approved using multivariate data analysis such as the principal component analysis (PCA). In this case, aromatic in-plane deformation FTIR absorption signals (at about 1160 cm^−1^) corresponding to the monolignol substitution pattern could be assigned to differentiate between stem and leaf lignins. Due to HSQC NMR results, there are also differences in monolignol composition for the two *M*. x *giganteus* samples (Gig17, Gig34). In comparison to beech wood-based lignin obtained from organosolv pulping at similar conditions, *Miscanthus* lignins possess a lower molecular weight and a very narrow polydispersity (<1.5 for *M*. genotypes versus >2.5 for beech wood lignins) due to a more homogeneous structure of grass versus wood-derived lignins. The obtained results emphasize the potential of *Miscanthus* crop as a low-input resource and agricultural feedstock for sustainable material production.

## Figures and Tables

**Table 1:** Abundance of linkages in lignins of soft and hard wood [61] and *Miscanthus* grasses [62,63] including KOH-extractable and non-KOH-extractable in %.

Linkage	Hard Wood H/G/S traces/25–50/50–75	Soft Wood H/G/S 0.5–3.4/90–95/0–1	*Miscanthus* H/G/S 24/49/27
β-O-4‘	50–65	43–50	93
A-O-4‘	4–8	6–8	ns*
β-β‘	3–7	2–4	4
β-5‘	4–6	9–12	3
β-1‘	5–7	3–7	traces
4-O-5’	6–7	4	ns *
5-5’	4–10	10–25	ns *

* ns: not specified.

**Table 2:** Compositional analysis data (according to NREL protocol) of *Miscanthus* x *giganteus* (Gig17, Gig34) and biomasses of leaf versus grasses, harvested in April 2015. AIL: acid-insoluble lignin. ASL: acid-soluble lignin. AIR: acid-insoluble residuals. AISA: acid-insoluble ash.

Genotype Harvest Time Portion	Gig17 April Leaf	Gig34 April Leaf	Gig17 April Stem	Gig34 April Stem
AIL (%)	20.65 ± 0.47	21.06 ± 0.31	21.18 ± 0.01	20.97 ± 0.43
ASL (%)	5.06 ± 0.30	4.13 ± 0.05	4.71 ± 0.26	4.31 ± 0.02
AIR (%)	21.83 ± 0.51	22.14 ± 0.42	22.46 ± 0.22	22.31 ± 0.60
Total lignin (%)	25.52 ± 0.54	25.32 ± 0.48	26.00 ± 0.36	25.25 ± 0.39
AISA (%)	1.18 ± 0.10	1.08 ± 0.22	1.29 ± 0.11	1.01 ± 0.05
Glucan (%)	44.79 ± 1.50	44.98 ± 2.30	50.02 ± 0.63	50.49 ± 0.87
Xylan (%)	28.37 ± 1.77	29.52 ± 0.63	27.41 ± 2.81	26.23 ± 0.49
Galactan (%)	0.00 ± 0.00	0.00 ± 0.00	0.00 ± 0.00	0.00 ± 0.00
Arabinan (%)	3.11 ± 0.05	4.11 ± 1.63	1.89 ± 0.51	2.04 ± 0.34
Mannan (%)	0.00 ± 0.00	0.00 ± 0.00	0.00 ± 0.00	0.00 ± 0.00
Dry matter (%)	92.55	91.25	92.18	92.16
Total ash (%)	4.53	6.82	2.50	3.07

**Table 3:** FTIR signals and corresponding assignment for a lignin isolated from *M.* x *giganteus* (Gig17, stem/leaf mixture).

Number	Wave Number [cm^−1^]	Functional Group	Assignment
1	3428 ± 60	O-H	Stretching
2	2926 ± 11	C-H	Stretching
3	2850 ± 6	C-H	Stretching
4	1708 ± 11	C=O	Stretching
5	1655 ± 4	C=O	Stretching
6	1605 ± 12	Aromatic ring	Symmetric Stretching
7	1514 ± 6	Aromatic ring	Antisymmetric Stretching
8	1460 ± 4	C-H	Antisymmetric Deformation
9	1424 ± 4	C-H in O-CH_3_	Antisymmetric Deformation (S mode)
10	1398 ± 4	C-H	Bending C-H in-plane
11	1371 ± 15	Aromatic skeleton	Deformation
12	1331 ± 9	Aromatic skeleton; C-O	Skeleton Stretching (S mode)
13	1267 ± 2	Aromatic skeleton; C-O	Skeleton Stretching (G mode)
14	1225 ± 12	C-C; C-O; C=O	Stretching (G mode)
15	1166 ± 10	C-H (in G)	Stretching
16	1124 ± 2	Aromatic C-H	In-plane Deformation
17	1033 ± 2	Aromatic C-H	In-plane Deformation
18	834 ± 4	Aromatic C-H	Out-of-plane Deformation (S mode)

**Table 4:** SEC results (detected via refractive index (RI) and ultraviolet (UV) detectors) including the number average (Mn) and weight average (Mw) molecular weight and polydispersity (PD) of lignin obtained from *Miscanthus* x *giganteus* mixtures (Gig17M, Gig34M), leaves (Gig17L, Gig34L), and stems (Gig17S, Gig34S).

SEC Results	Detector	Gig17M	Gig34M	Gig17L	Gig34L	Gig17S	Gig34S
*M*_n (g·mol^−1^)_	RI	1138.80	1139.10	967.13	960.96	1314.40	1206.30
	UV	1077.70	1084.40	904.49	931.43	1168.90	1120.00
*M*_w (g·mol^−1^)_	RI	2041.40	2146.40	1758.70	1800.10	2385.00	2098.70
	UV	1983.40	2050.90	1691.60	1745.00	2216.30	2033.90
PD	RI	1.79	1.88	1.82	1.87	1.81	1.74
	UV	1.84	1.89	1.87	1.87	1.90	1.82

**Table 5:** TGA results including first and second mass loss (ML), residual mass (RM), and decomposition temperature (T_d_) for two *M*. x *giganteus* lignins. Mixtures (Gig17M, Gig34M), stem-derived lignins (Gig17S, Gig34S), and leaf-derived lignins (Gig17L, Gig34L).

Sample	First ML (120 °C)	Second ML (350 °C)	RM	T_d_
Gig17M	0.86%	75.88%	23.26%	358.2 °C
Gig17L	0.91%	64.98%	34.11%	354.8 °C
Gig17S	0.42%	65.48%	34.10%	381.6 °C
Gig34M	0.54%	73.79%	25.67%	360.0 °C
Gig34L	0.72%	71.41%	27.87%	336.1 °C
Gig34S	4.60%	84.08%	11.32%	379.2 °C

**Table 6:** Pyrolysis-GC/MS fragments of the sample Gig17M and assignment to H, G, and S units.

Peak No.	Retention Time (min)	Amount (%)	Name	Assignment
1	9.49	3.77	Phenol	H
2	11.36	10.07	Guaiacol	G
3	11.65	1.83	*p*-Cresol	H
4	13.32	0.70	Creosol	G
5	13.67	1.27	4-Ethylphenol	H
6	13.72	12.92	Creosol	G
7	15.55	0.91	4-Ethylguaiacol	G
8	15.69	22.08	Coumaran	H
9	16.874	8.17	4-Hydroxy-2-methylacetophenone	H
10	17.66	1.67	Eugenol	G
11	17.87	5.68	Syringol	S
12	19.00	1.55	Eugenol	G
13	20.13	6.80	4-Methylsyringol	S
14	20.24	6.23	(*E*)-Isoeugenol	G
15	21.05	1.91	Vanillin	G
16	24.26	1.68	4-Allylsyringol	S
17	25.95	1.56	(*E*)-4-Propenylsyringol	S
18	28.20	6.91	(*E*)-4-Propenylsyringol	S
19	32.03	2.50	Syringaldehyde	S
20	34.70	1.78	Acetosyringone	S

**Table 7:** Monolignol ratio of lignins according to pyrolysis GC/MS data of *M*. x *giganteus* samples (in %): Mixtures (Gig17M, Gig34M), leaves (Gig17L, Gig34L), and stems (Gig17S, Gig34S).

Sample	H (%)	G (%)	S (%)
Gig17M	37.13	35.96	26.91
Gig17L	46.35	45.82	7.83
Gig17S	35.11	32.94	31.95
Gig34M	43.79	29.50	26.71
Gig34L	46.30	39.25	14.45
Gig34S	35.06	38.55	26.39

**Table 8:** HSQC 2D NMR data for an *M*. x *giganteus* stem/leaf mixture (Gig17M).

Number	Integral (rel)	δ ^1^H (ppm)	δ ^13^C (ppm)	Name	Assignment
1	0.0190	3.45	52.88	B	B
2	0.0254	3.06	53.34	C	C
3	0.7089	3.56	59.66	A	A
4	0.1322	3.23	59.71	B	B
5	0.1932	3.68	62.73	A	A
6	0.0321	3.82	71.00	C	C
7	0.0441	4.17	70.76	C	C
8	0.0561	4.74	70.95	A	A
9	0.1541	4.86	71.37	A	A
10	0.0435	4.47	79.78	A	A
11	0.1160	4.31	83.63	A	A
12	0.0361	4.64	84.75	C	C
13	0.1106	4.07	85.77	A	A
14	0.0626	5.45	86.79	B	B
15	0.7042	6.73	103.42	S 2/6	S
16	0.0411	6.99	103.24	S 2/6	S
17	0.0337	7.33	103.79	S 2/6	S
18	0.4662	6.96	109.88	G 2	G
19	0.1192	6.30	113.46	D	D
20	1.0000	6.71	114.80	G 5	G
21	0.1827	6.79	118.61	G 6	G
22	0.1588	7.12	127.35	H 2/6	H
23	0.4032	7.48	129.07	H 2/6	H
24	0.1315	7.43	143.84	D	D

**Table 9:** Ratio of the monomer units (H, G, S) in lignins obtained from *M.* x *giganteus*. Stem/leaf mixtures (Gig17M, Gig34M), stems (Gig17S, Gig34S), and leaves (Gig17L, Gig34L).

Sample	H	G	S
Gig17M	23.03	45.05	31.92
Gig17L	13.65	67.24	19.11
Gig17S	15.77	57.67	26.56
Gig34M	21.17	53.54	25.29
Gig34L	14.87	63.43	21.70
Gig34S	19.07	54.80	26.13

**Table 10:** Ratio of the most abundant linkages in lignins obtained from *M*. x *giganteus*. Stem/leaf mixtures (Gig17M, Gig34M), stems (Gig17S, Gig34S), and leaves (Gig17L, Gig34L).

Sample	A (β-Aryl Ether) %	B (Phenylcoumaran) %	C (Resinol) %	D (Unsaturated Ester) %
Gig17M	61.84	9.56	6.16	22.43
Gig17L	65.31	9.43	4.35	20.91
Gig17S	58.14	9.37	8.18	24.31
Gig34M	62.62	7.70	8.00	21.68
Gig34L	66.41	10.10	4.92	18.57
Gig34S	54.73	8.22	7.28	29.77

**Table 11:** SEC set up and parameters.

Component	Description	Parameter
Eluent	THF p.a.	HPLC-grade
Pump	Agilent 1100 Series	Flowrate: 1.000 mL min^−1^
Injector	Rheodyne 7725i sample loop	Injection volume: 60 µL
Oven	Agilent 1100 Series	Temperature: 35 °C
Column	1× PSS SDV 8 × 50 mm pre-column	Particle size: 5 µm
	2× PSS SDV 8 × 300 mm Linear M 5µ	Particle size: 5 µm Molar weight range: 50–10,000,000 g mol^−1^
Detector	Agilent 1100 Series VWD	Wavelength: 280 nm
Calibration	PSS ReadyCal-Kit Polystyren	Calibration range: 376–2,570,000 g mol^−1^
